# Llgl1 prevents metaplastic survival driven by epidermal growth factor dependent migration

**DOI:** 10.18632/oncotarget.11320

**Published:** 2016-08-17

**Authors:** Erin Greenwood, Sabrina Maisel, David Ebertz, Atlantis Russ, Ritu Pandey, Joyce Schroeder

**Affiliations:** ^1^ Department of Molecular and Cellular Biology, University of Arizona, Tucson, Arizona; ^2^ Arizona Cancer Center, University of Arizona, Tucson, Arizona; ^3^ BIO5 Institute, University of Arizona, Tucson, Arizona; ^4^ Genetics Program, University of Arizona, Tucson, Arizona; ^5^ Cancer Biology Program, University of Arizona, Tucson, Arizona; ^6^ Cell and Molecular Medicine, University of Arizona, Tucson, Arizona

**Keywords:** polarity, migration, Llgl1, epidermal growth factor receptor, TAZ

## Abstract

We have previously demonstrated that Llgl1 loss results in a gain of mesenchymal phenotypes and a loss of apicobasal and planar polarity. We now demonstrate that these changes represent a fundamental shift in cellular phenotype. Llgl1 regulates the expression of multiple cell identity markers, including CD44, CD49f, and CD24, and the nuclear translocation of TAZ and Slug. Cells lacking Llgl1 form mammospheres, where survival and transplantability is dependent upon the Epidermal Growth Factor Receptor (EGFR). Additionally, Llgl1 loss allows cells to grow in soft-agar and maintain prolonged survival as orthotopic transplants in NOD-SCIDmice. Lineage tracing and wound healing experiments demonstrate that mammosphere survival is due to enhanced EGF-dependent migration. The loss of Llgl1 drives EGFR mislocalization and an EGFR mislocalization point mutation (P667A) drives these same phenotypes, including activation of AKT and TAZ nuclear translocation. Together, these data indicate that the loss of Llgl1 results in EGFR mislocalization, promoting pre-neoplastic changes.

## INTRODUCTION

Epithelial cells are regulated by apicobasal polarity complexes that provide an asymmetric cell structure, regulate growth and survival, migration and invasion, and differentiation [1]. Polarity is established first by the Crumbs complex creating the apical membrane, followed by the Par complex establishment of the apical-lateral border, and the Scribble complex defining the basolateral domain. The Scribble complex consists of Scribble, Discs large (Dlg), and Lethal giant larvae (LGL). Humans have two orthologs, Llgl1 and Llgl2, also known as Hugl1 and Hugl2 [2]. Llgl1 is down regulated in many cancers, including colorectal, endometrial, hepatocellular carcinoma, malignant melanomas, and breast cancer [2–6]. Llgl1 loss correlates with lymph node metastases, advanced stage, and poor prognosis [3–5]. In addition, deletion mutants of Llgl1 encoding truncated proteins correlate with advanced disease and tumorigenicity in mice [5]. Advanced metastatic disease is frequently associated with stem cell characteristics, in that both stem cells and highly metastatic cancers exhibit drug resistance, migratory capacity, self-renewal, and the ability to survive and differentiate into new tissues [7]. A number of molecular features associated with polarity loss are similarly found in both stem cells and metastatic cancer.

Genetic interaction studies demonstrate that Lgl can regulate the transcription factor Yorkie [Transcription co-Activator with a PDZ-binding domain (TAZ) and the Yes-Associated Protein (YAP) in mammals] [8]. This transcription factor family is a known modulator of epithelial differentiation [9–12] and their activity may drive stem-cell like phenotypes [13–15]. Nuclear translocation of YAP and TAZ can also promote breast cancer stem cell properties including self-renewal, migration, and tumor-initiating abilities [16–18]. In addition, YAP and TAZ expression is elevated in advanced breast cancer and inversely correlates with metastasis-free survival [16]. Nuclear translocation of YAP and TAZ can be driven by the Epidermal Growth Factor Receptor family member ErbB-4 and/or EGFR-dependent activation of the MAP Kinase pathway [17, 19, 20]. In fact, autocrine loops between the EGFR pathway and YAP activation have been identified in ovarian cancer and breast epithelium [21, 22].

While the EGFR family of receptors and ligands have been shown to activate the YAP/TAZ transcription factors in some studies, they are also established drivers of survival, migration and growth *via* the RAS/MAP Kinase and AKT pathways. One facet of pathway activation is intracellular localization of the receptors, as changes to intracellular localization of EGFR have been shown to play key roles in the activation of signal transduction cascades. In fact, EGFR preferentially activates both the RAS/MAP Kinase and AKT survival pathways when in endosomes compared to the plasma membrane [23]. These studies indicate that changes to EGFR localization may be a key event in EGFR-driven events such as neoplasia and metastasis.

The cell of origin of heterogeneic metastases has been linked to tissue stem cells, cancer stem cells, or transdifferentiating cells [7, 24]. Characteristics that define these cells include serial transplantation *in vitro* and *in vivo*, and the expression of a variety of surface markers, including CD44_hi_/CD24_lo_, CD44_hi_, and CD49f_lo_, among others [25–29]. Of note, TAZ nuclear translocation is known to potentiate EGFR signaling pathways, which in turn can increase CD44 transcription and stem cell characteristics [30, 31]. Due to the connection between receptor localization within a cell and its ability to activate these pathways, an epithelial population may possess a plasticity depending upon their state of polarity. To determine the role of Llgl1 in these events, we evaluated the effects of Llgl1 loss in normal (immortalized) breast epithelium. We discovered that Llgl1 regulates multiple cellular phenotypes resembling a highly migratory state capable of surviving transplantation as mammospheres and in the mammary gland of immunocompromised mice. These effects are dependent upon the mislocalization of EGFR and its corresponding effects on EGFR driven activation of AKT and TAZ.

## RESULTS

### Llgl1 regulates cell morphology

**Figure 1:**
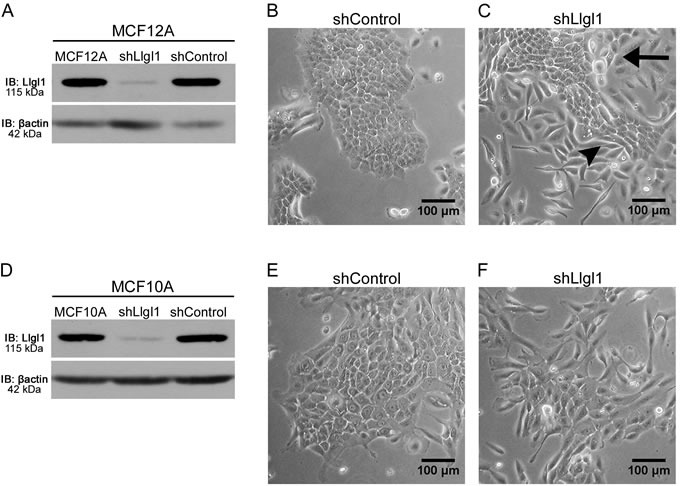
Llgl1 expression regulates cell morphology Stable knockdown was established in MCF10A and MCF12A cells with transduction of Llgl1 or control shRNA lentiviral particles. **A.** and **D.** Protein lysates were analyzed by immunoblot using the antibodies: anti-Llgl1 and anti-βactin. **B.** and **C.** Brightfield images of MCF12A cells comparing shControl to shLlgl1. **E.** and **F.** Brightfield images of MCF10A cells comparing shControl to shLlgl1. The arrow indicates cobblestone and the arrowhead indicates mesenchymal morphologies.

Loss of Llgl1 in MCF10A and HMEC cells has been shown previously to result in a mesenchymal phenotype [32]. To further evaluate this biology, Llgl1 expression was knocked down in both MCF12A and MCF10A cells, two different spontaneously immortalized breast epithelial cell lines. Cells were transduced with either a control shRNA (shControl) or with a shRNA designed to silence Llgl1 expression (shLlgl1), that was optimized previously using 5 different shRNA targets [32] (Figure 1A and 1D, Supplemental Figure 1). In MCF10A cells, shLlgl1 resulted in an increase in mesenchymal morphology (Figure 1F). Alternatively, in MCF12A shLlgl1 cells, two distinct cell morphologies can be observed, including cobblestone and mesenchymal (Figure 1B and 1C arrow vs arrow-head respectively). Considering the mixed phenotype observed upon Llgl1 knockdown (Figure 1C), we next set out to determine the identity of these two cell populations.

### Llgl1 regulates expression of cell lineage markers

**Figure 2:**
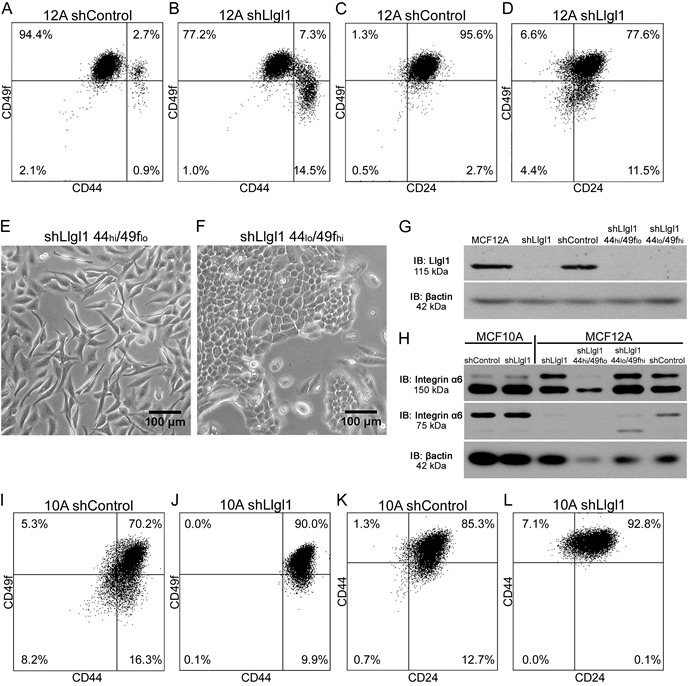
Llgl1 regulates expression of cell lineage markers **A.**-**D.**, **I.**-**L.** Cells were incubated with the indicated cell lineage marker and sorted by FACS. **A.**-**D.** MCF12A shControl vs shLlgl1 (under normal growth conditions) were incubated with anti-CD49f-PE, anti-CD44-APC, and/or anti-CD24-FITC. **E.** and **F.** MCF12A shLlgl1 were sorted based on CD44/CD49f expression, CD444_hi_/CD49f_lo_ (E) and CD44_lo_/CD49f_hi_ (F). **G.** Protein lysates were isolated and analyzed by immunoblot using anti-Llgl1 and anti-βactin antibodies. **H.** Protein lysates were analyzed by immunoblot using anti-Integrin α6 and anti-βactin. (I-L) MCF10A shControl vs shLlgl1 (under normal growth conditions) were incubated with anti-CD49f-PE, anti-CD44-APC, and/or anti-CD24-FITC.

To investigate the differences caused by the loss of Llgl1 and the different populations that are observed, we evaluated the cells using breast cancer stem cell markers CD44, CD49f, and CD24 [25–29]. Analysis of the parental MCF12A and shControl transduced cells revealed a consistent CD44_lo_/CD49f_hi_/CD24_hi_ phenotype (data not shown and Figure 2A and 2C). However, the shLlgl1 transduced cells showed two populations, a CD44_lo_/CD49f_hi_ population and a CD44_hi_/CD49f_lo_ population (Figure 2B). In addition, shLlgl1 cells also displayed a decrease in CD24 compared to the shControl (Figure 2D). To evaluate if the shLlgl1-induced populations were indicative of the different phenotypic morphologies observed, we sorted the shLlgl1 transduced MCF12A cells based on CD44_hi_/CD49f_lo_ expression. The shLlgl1 cells that expressed CD44_hi_/CD49f_lo_ were morphologically mesenchymal (Figure 2E), while shLlgl1 cells that expressed CD44_lo_/CD49f_hi_ resembled the normal cobblestone morphology (Figure 2F). Loss of Llgl1 expression was confirmed in these sorted populations, as was Integrin α6 expression (Figure 2G-2H). While the full length protein (150 kDa) is lacking in the CD49f_lo_ population as expected, we also observed a loss of cleaved Integrin α6 (75 kDa) expression in all Llgl1 knockdown MCF12A lines (Figure 2H).

Analysis of the MCF10A shLlgl1 transduced cells compared to the parental and shControl cells also showed a reduction in CD24 and an increase in CD44 (data not shown and Figure 2I-2J) but no separation into two distinct populations, echoing the single phenotypic morphology seen in tissue culture. While MCF12A and MCF10A cells are both immortalized breast epithelial cells, these differences in lineage expression may represent the fact that each line was independently derived from reduction mammoplasty, and likely contain different stem cell populations [33].

### Llgl1 loss drives EGFR-dependent mammosphere formation

**Figure 3:**
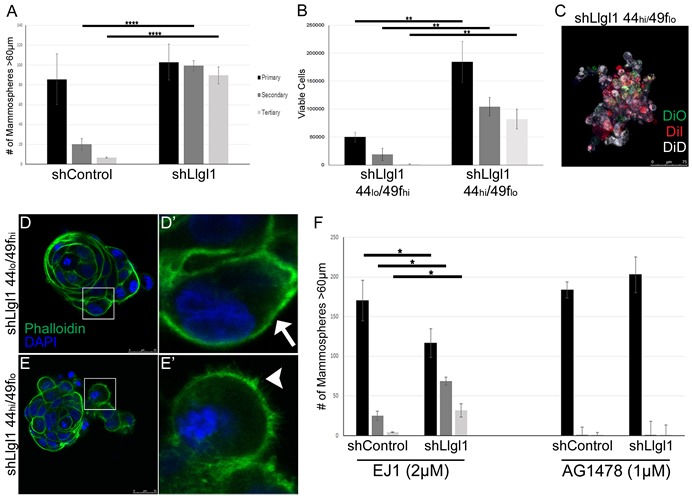
Loss of Llgl1 induces EGF-dependent mammosphere formation **A.**-**F.** Mammosphere assays were performed using cells generated as described in Figure 1 and Figure 2. **A.** Mammosphere formation with EGF treatment (20ng/mL) was quantified by counting mammospheres greater than 60μm (3 replicates per treatment group, each experiment was performed 3 times). **B.** Due to coalescing mammospheres in shLlgl1 CD44_hi_/CD49f_lo_ mammopshere formation was determined by counting viable cells at each time point (3 replicates per treatment group, each experiment was performed 3 times). **C.** MCF12A shLlgl1 CD44_hi_/CD49f_lo_ cells from primary mammospheres were labeled with the lipophilic tracer dyes Di-O, Di-I, or Di-D. **D.**-**E.** Mammospheres from shLlgl1 CD44_lo_/CD49f_hi_ and CD44_hi_/CD49f_lo_ populations were incubated with Alexa Fluor 488 phalloidin (green) and DAPI (blue). The arrow indicates smooth cortical actin (D′) and the arrowhead indicates invasive cortical actin (E′). **F.** Mammospheres were treated with either 2μM EJ1 or 1μm AG1478. All primary passages are shown in black, secondary passages in dark gray, and tertiary passages in light gray. Error bars show ± standard deviation. *P < 0.05, **P < 0.01, ****P < 0.0001.

As alterations in CD44, CD49f, and CD24 are markers of stem cells, we next evaluated the ability of these cells to form mammospheres over a primary, secondary, and tertiary passage (Figure 3). MCF10A and MCF12A shControl versus shLlgl1 cells were evaluated and a significant increase in the ability of cells to form mammospheres and to grow through secondary and tertiary passages was observed (Supplemental Figure 2 and Figure 3A). To further evaluate the role of stem cell marker expression in mammosphere formation, MCF12A shLlgl1 cells were FACS sorted based on the CD44_hi_/CD49f_lo_ and CD44_lo_/CD49f_hi_ profiles, and revealed that shLlgl1 CD44_hi_/CD49f_lo_, but not CD44_lo_/CD49f_hi_ cells were able to form mammospheres and continue to form them over three serial passages (Figure 3B).

Mammospheres can form from either a single stem-like cell or from multiple cells with enhanced survival capacity migrating together, phenotypes that can be verified by lineage tracing [34]. After primary mammosphere formation was complete, MCF12A shLlgl1 CD44_hi_/CD49f_lo_ cells were dissociated, divided into three groups, stained with Di-O, Di-I, or Di-D, replated for secondary passage, and imaged. This analysis revealed that all mammospheres contained cells of multiple colors (Figure 3C). These data indicate that the mammospheres did not form from a single cell, as a stem cell would generate, but from cells migrating together. Although the mammospheres are not forming from a single stem cell-like precursor, they are able to survive and grow significantly better in the absence of Llgl1 as compared to the controls. In addition, invasive cell edges were seen in the CD44_hi_/CD49f_lo_ but not the CD44_lo_/CD49f_hi_ population, which is highlighted by the structure of cortical actin (Figure 3D′ arrow vs 3E′ arrowhead).

As EGF treatment can induce migration and mammosphere formation, we next set out to determine if mammosphere formation was EGF dependent. Evaluation of MCF12A shControl vs shLlgl1 cells revealed that mammosphere formation was significantly inhibited in the presence of either EGFR kinase inhibitor (AG1478) or EGFR dimerization inhibitor (EJ1) [35] (Figure 3F). Inhibition of EGFR dimerization or kinase activity, while inducing cell growth in the primary passage, reduced mammopshere formation in the secondary and tertiary passages (compare Figure 3A to Figure 3F). These data indicate that both a loss of Llgl1 and EGFR activation are required for mammosphere survival. Note that the dimerization inhibitor impacts a number of kinase independent functions for EGFR including Calmodulin activation and ROS generation [35]. These additional activities may account for the activity observed with the dimerization inhibitor versus the kinase inhibitor (Figure 3F).

### Llgl1 loss drives EGFR mislocalization and novel signal transduction

**Figure 4:**
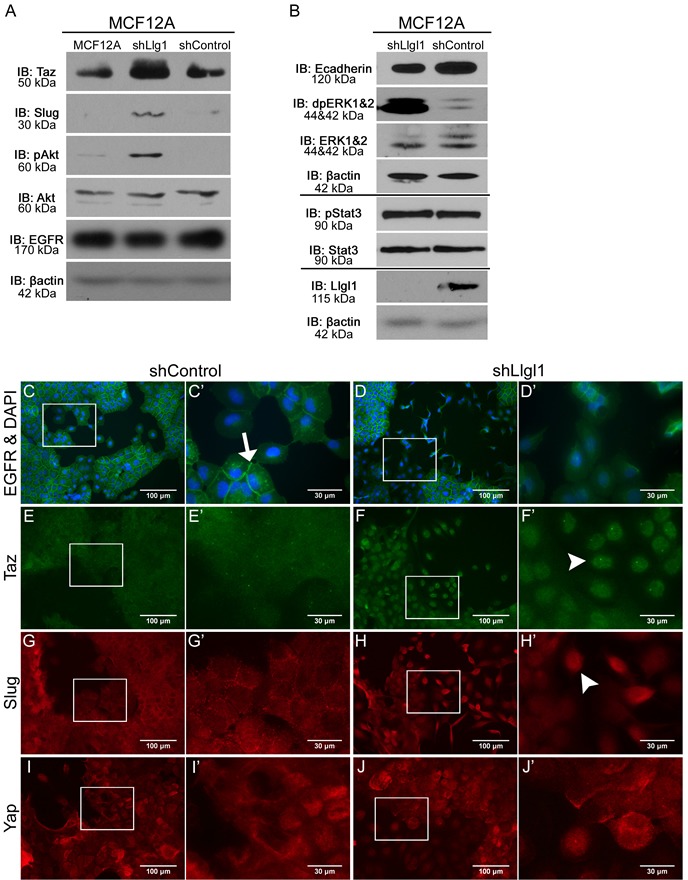
Llgl1 loss drives EGFR mislocalization and novel signal transduction **A.** and **B.** Protein lysates were collected from MCF12A parental, shControl, and shLlgl1 cells and analyzed by immunoblot using the antibodies: anti-TAZ, anti-Slug, anti-pAkt, anti-Akt, anti-EGFR, anti-E-cadherin, anti-dpERK1/2, anti-ERK1/2, anti-pStat3, anti-Stat3, anti-Llgl1, and anti-βactin. Immunoblots against anti-βactin are shown for each set of lysates. **C.**-**J.** MCF12A shControl and shLlgl1 cells were grown on plastic and (C-D) serum starved overnight or (E-J) in normal growth conditions then evaluated for localization of the indicated proteins. Cell were incubated with either anti-EGFR 1005, anti-TAZ, anti-SLUG, or anti-YAP antibodies and mounted (C-D) with DAPI or (E-J) without DAPI. Arrows indicate membrane localization and arrowheads indicate nuclear localization. C′, D′, E′, F′, G′, H′, I′, and J′ panels represent increased magnifications of C, D, E, F, G, H, I, and J panel insets.

EGFR activity is known to drive multiple signal transduction pathways as well as induce a transient loss of epithelial cell junctions [36]. Therefore, we evaluated MAP kinase activity (p42/44 ERK; designated dpERK), AKT activation, and localization of drivers of migratory phenotypes, including loss of E-cadherin, and gain of SLUG and TAZ expression. In the absence of Llgl1, we observed a significant increase in both dpERK and AKT, but not STAT3 activity (Figure 4A and 4B). A loss of E-cadherin, as well as increase in SLUG and TAZ, was also observed at the protein level in Llgl1 knockdown cells. In an effort to determine how EGFR pathways were being activated in the absence of the Llgl1 polarity program, we next evaluated EGFR localization. While MCF12A shControl cells display membrane-localized EGFR (Figure 4C and C′ arrow), shLlgl1 cells displayed two distinct EGFR localizations. While EGFR was membrane bound in cuboidal epithelium, it was diffuse throughout the cell in the mesenchymal population (Figure 4D and D′). EGFR activity is known to induce YAP/TAZ and SLUG nuclear translocation and activation under conditions leading to migration and stemness [20, 37]. We found that loss of Llgl1 results in nuclear translocation of TAZ and SLUG, but not YAP (Figure 4E-4J, arrowheads). These data indicate that Llgl1-dependent phenotypes differentially impact TAZ and YAP localization.

### Llgl1 loss promotes a migratory phenotype

**Figure 5:**
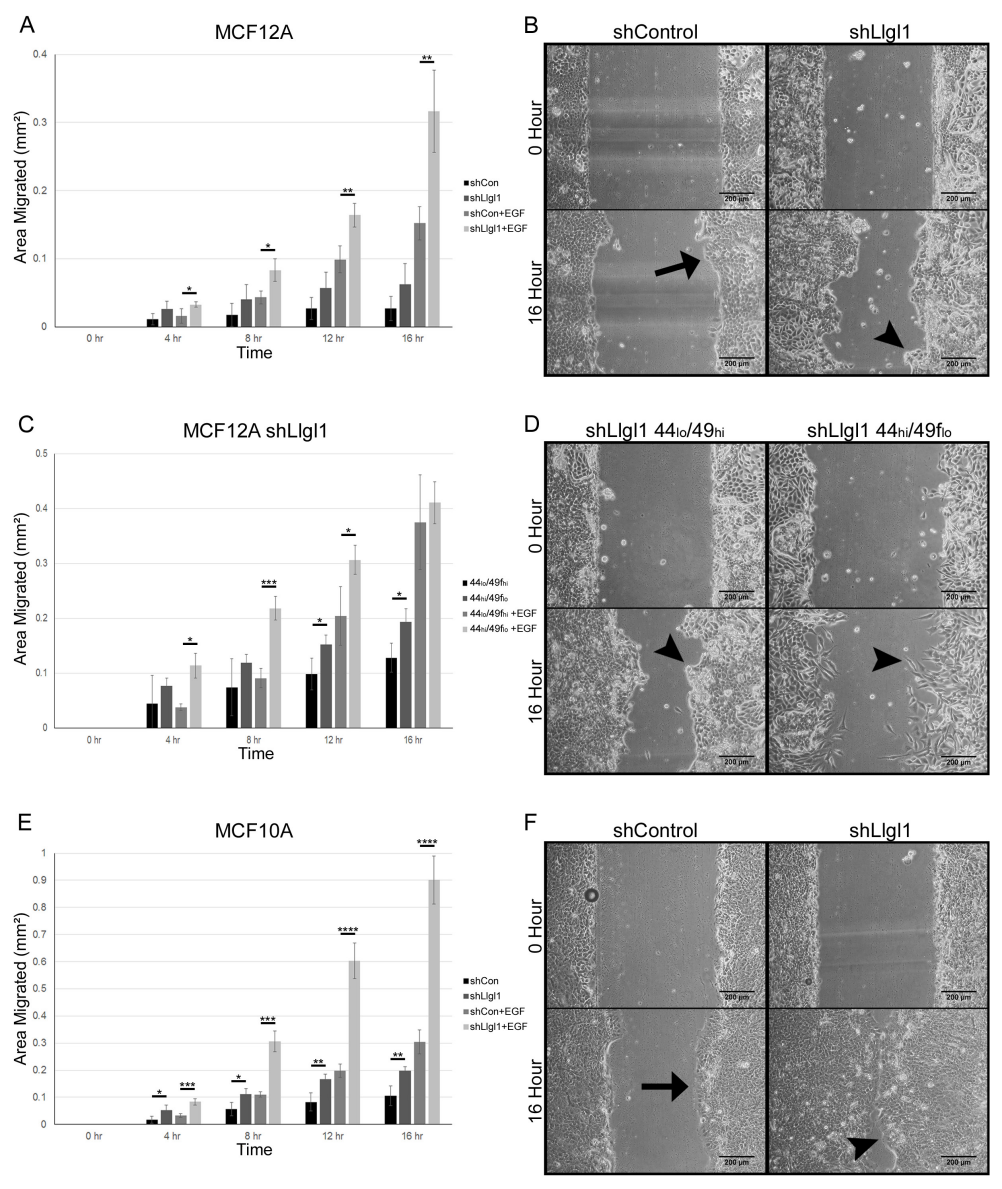
Llgl1 loss promotes a migratory phenotype **A.**-**F.** MCF12A and MCF10A control and Llgl1 knockdown cells and MCF12A shLlgl1 CD44_lo_/CD49f_hi_ and shLlgl1 CD44_hi_/CD49f_lo_ cells were generated as described in Fig. 1 and Fig. 2 and analyzed. Cells were grown to confluence, scratched, and then observed for wound healing migration in serum free media with either the absence of EGF (A, C, and E) or in the presence of EGF (20ng/mL). Migrating epithelial sheets are indicated by arrow, disorganized cellular groups and single cells are indicated by arrowheads. Error bars show ± standard deviation. *P < 0.05, **P < 0.01, ***P < 0.001, ****P < 0.0001.

To investigate the effects of Llgl1 expression on cellular migration we grew both MCF12A and MCF10A shControl and shLlgl1 cells and sorted MCF12A shLlgl1 populations to confluence and created a scratch in the epithelial sheet to stimulate wound healing (Figure 5). Absence of Llgl1 increased migration over control cells at all time points (Figure 5A and 5C compare shControl and shLlgl1). In addition, all cells responded to EGF with induced migration, but the Llgl1 knockdown cells had a significantly higher response to EGF (Figure 5A and 5C compare shControl+EGF and shLlgl1+EGF). Of note, shControl cells from both MCF10A and MCF12A tended to migrate en masse as a single epithelial sheet (Figure 5B and 5F arrows); alternatively, shLlgl1 cells tended to migrate as either single cells or disorganized groups (Figure 5B and 5F arrowheads). These differences in cell migration were further enhanced in the sorted MCF12A shLlgl1 populations, as the CD44_lo_/CD49f_hi_ cells migrated as a disorganized mass while the CD44_hi_/CD49f_lo_ cells migrated as single cells (Figure 5D). The CD44_hi_/CD49f_lo_ cells initially closed the wound faster but due to their lack of directionality, failed to close the wound completely (Figure 5C and Supplementary Video 1). To further demonstrate the EGFR dependence of this phenotype, similar experiments were performed with additional EGFR ligands, including Transforming Growth Factor alpha (TGFα) and Amphiregulin. Similar results were observed, with TGFα displaying the strongest pro-migratory effect of the two ligands (Supplemental Figure 3). Evaluation of cell growth between shControl and shLlgl1 cells found no significant change in the absence of EGF and either no significant change (MCF10As) or a decrease in cell growth (MCF12As) with EGF, indicating changes in migration are not due to increased cell number (Supplemental Figure 4).

### Mislocalization of EGFR drives pro-migratory and survival pathways

**Figure 6:**
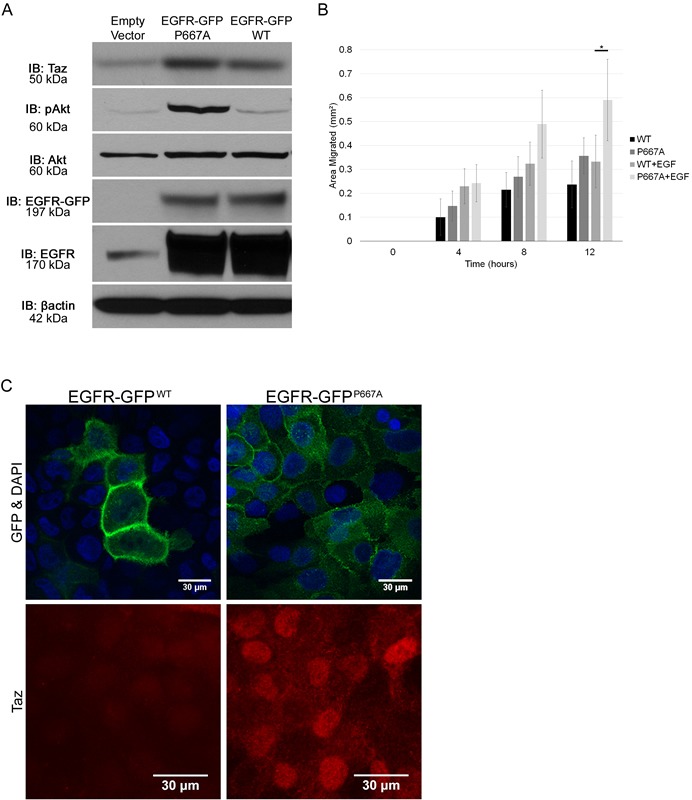
Mislocalization of EGFR drives pro-migratory and survival pathways **A.** Protein lysates were collected from MDCK cells transfected with an Empty Vector, an EGFR-GFP^P667A^ vector, or an EGFR-GFP^WT^ vector, and analyzed by immunoblot using the antibodies: anti-TAZ, anti-pAKT, anti-AKT, anti-EGFR, and anti-βactin. The EGFR-GFP blot indicates the GFP tagged EGFR induced via vector transfection while the EGFR blot indicates all EGFR present. **B.** EGFR-GFP^WT^ and EGFR-GFP^P667A^ transfected MDCK cells were grown to confluence, scratched, and then observed for wound healing migration in serum free media in either the absence of EGF or in the presence of EGF (20ng/mL). Error bars show ± standard deviation. *P < 0.05. **C.** MCF12A cells were transfected with EGFR-GFP^WT^ or EGFR-GFP^P667A^ vector, grown on plastic, serum starved overnight, mounted with DAPI and evaluated for localization of EGFR-GFP and TAZ using an anti-GFP and anti-TAZ antibody, respectively.

We have determined that Llgl1 regulates mammosphere formation and migration, and that these events are EGFR dependent. Further, Llgl1 loss induces AKT and MAP Kinase activation, downstream mediators of EGFR activity. As Llgl1 induces EGFR intracellular localization, and intracellular EGFR localization is reported to be tied to activation of these signaling cascades, we next set out to determine if mislocalization of EGFR itself could drive these same effects. To test this hypothesis, we expressed an EGFR basolateral targeting domain point mutation (P667A) that redirects EGFR throughout the plasma membrane and intracellular localizations, including vesicles [38]. MDCK and MCF12A cells were transfected with either wild type EGFR-GFP (EGFR-GFP^WT^) or mutant P667A EGFR-GFP (EGFR-GFP^P667A^) and evaluated for EGFR localization and activation of signal transduction pathways. We found that this mutation does in fact result in EGFR mislocalization, and this is associated with increased TAZ nuclear translocation as compared to EGFR-GFP^WT^ (Figure 6C). Under similar levels of expression (Figure 6A) we found that AKT activation and TAZ expression were both significantly increased in the EGFR-GFP^P667A^ over the EGFR-GFP^WT^ (Slug is not expressed in parental MDCK cells). To determine if mislocalization of EGFR also drives an increase migration, we performed migration assays as described in Figure 5. We observed a similar, EGF-dependent increase in migration in EGFR-GFP^P667A^ compared to EGFR-GFP^WT^. Overall, these data indicate that EGFR mislocalization can drive a set of pro-migratory survival pathways.

### Llgl1 loss promotes cell piling and increased survival in soft agar and mammary fat pads

**Figure 7:**
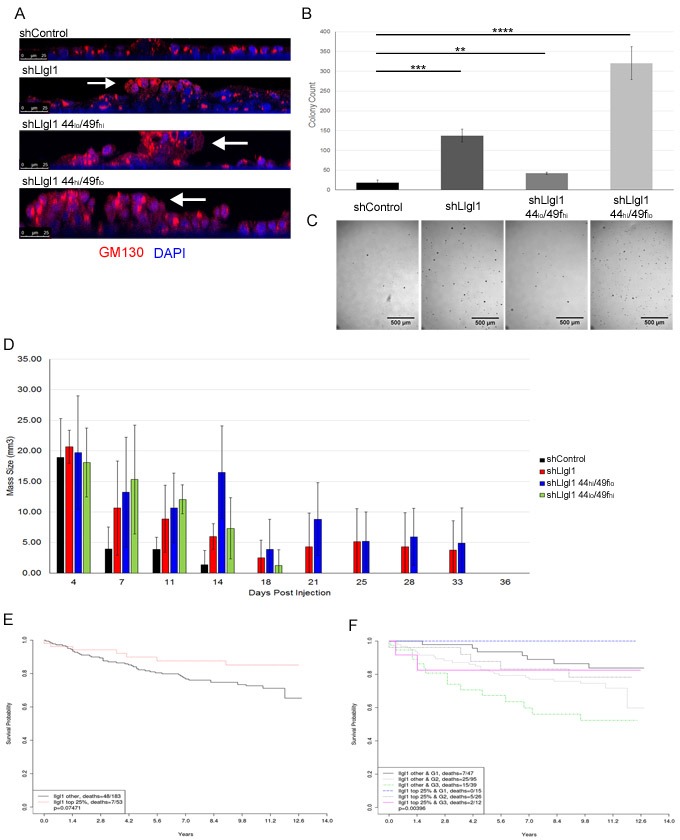
Llgl1 loss promotes cell piling and increased survival in soft agar and mammary fat pads and its expression correlates with increased survival in metastatic breast cancer patients MCF12A shControl, shLlgl1, shLlgl1 CD44_hi_/49f_lo_, and shLlgl1CD44_lo_/49f_hi_ cells were grown (A) on filters, (B and C) in soft agar, or (D) in the mammary fat pads of NOD-SCID mice. **A.** Cells were grown on transwell filters in normal growth media and probed with anti-GM130 antibody and DAPI. Cells were then imaged on the confocal microscope in the Z plane, arrows indicate polyp formation. **B.** and **C.** Cells were plated in soft agar, allowed to grow for 13 days, visualized and enumerated with ImageJ. Representative images (C) are shown beneath quantifications (B). 3 replicates per treatment group, experiment repeated twice. **D.** 500,000 MCF12A shControl, shLlgl1, shLlgl1 CD44_hi_/CD49f_lo_, and shLlgl1 CD44_lo_/CD49f_hi_ cells were suspended in matrigel and injected into the mammary fat pad of NOD-SCID mice. Cell masses were palpated and measured every 3-5 days, shControl *n* = 3, *n* = 4 for all other groups. Kaplan-Meier analysis of GSE3494 dataset, (E) N = 236 and (F). N = 234, were analyzed for Llgl1 expression and divided into two groups, top 25% expression levels of Llgl1 and the remaining 75% of lower expression. **E.** Survival curves show that loss of Llgl1 in breast cancer tumors correlates with lower survival probability. **F.** Tumor samples were further grouped by elston grades (G1 = grade 1 tumor, G3 = grade 3 tumor) and survival curves for these subgroups are shown (Bottom 75% Llgl1 expression in Grade 3 tumors = dotted green line, top 25% Llgl1 expression in Grade 3 tumors = solid pink line).

To further evaluate the effects of Llgl1 loss on survival and pre-neoplastic pathways, MCF12A shControl, shLlgl1, shLlgl1 CD44_hi_/CD49f_lo_, and shLlgl1 CD44_lo_/CD49f_hi_ were grown on filters to allow cells to polarize and form epithelial sheets. While control cells displayed apicobasal polarity (Figure 7A, top panel), the remaining three cell types lacking Llgl1 formed multiple layers and grew into polyps along the vertical axis (Figure 7A bottom 3 panels, arrows). Additionally, when grown in soft agar, shControl cells had very low colony growth (Figure 7B and 7C, left column and image), while the loss of Llgl1 expression induced a significant increase in colony formation and growth (Figure 7B and 7C, second column and image). Furthermore, shLlgl1 cells sorted for CD44_hi_/CD49f_lo_ expression exhibited an even greater ability to form colonies (Figure 7B and 7C, forth column and image).

To investigate the effect of Llgl1 expression on tumor forming capabilities *in vivo*, shControl, shLlgl1, shLlgl1 CD44_hi_/CD49f_lo_, and shLlgl1 CD44_lo_/CD49f_hi_ cells were injected in the mammary fat pads of NOD-SCID mice with matrigel into the mammary fat pads of NOD-SCID mice and evaluated for survival (Figure 7D). While all cell types were able to grow initially, by two weeks no shControl cells survived and by three weeks shLlgl1 CD44_lo_/CD49f_hi_ cells no longer survived. Alternatively, shLlgl1 and shLlgl1 CD44_hi_/CD49f_lo_ cells both survived an additional two weeks (Figure 7D). This enhanced persistence in the absence of Llgl1 further demonstrates the impact of Llgl1 on the ability of a cell to survive in three dimensions and corresponds with increased activation of AKT.

### Llgl1 expression correlates with prolonged survival in metastatic cancer patients

As loss of Llgl1 enhanced survival and migration of immortalized epithelial cells, we next evaluated Llgl1 expression in patient samples. First, 11 Patient-Derived Xenograft (PDX) lines were evaluated for Llgl1 and EGFR expression (Supplemental Figure 5). The eleven lines tested included triple negative and Her2 positive breast cancers of grade 2 or higher, and we observed that Llgl1 loss was associated with increased EGFR expression. EGFR expression, high CD44 expression, and migration have all been linked to decreased patient survival [39–44]. We therefore analyzed a publicly available GEO data set (GSE3494) with 236 breast cancer patients for Llgl1 expression and long term survival (Figure 7E). Patients with highest Llgl1 expression trended towards the highest overall survival (Figure 7E red line) compared with patients who had lower levels of Llgl1 expression (Figure 7E black line). This effect was significantly enhanced by stratification of patients into grade 1 and grade 3 tumors, which separates patients based on metastatic progression (Figure 7F). Overall, these data indicate that Llgl1 expression may significantly affect tumor progression in breast cancer patients.

## DISCUSSION

In the current report we have demonstrated that Llgl1 loss alters the expression of CD44, CD49f, and CD24, and allows for growth in soft agar, survival in mammary fat pads, and EGFR-dependent survival in mammospheres. In addition, Llgl1 loss drives the mislocalization of EGFR and activation of AKT, MAP Kinase and TAZ. Further analysis demonstrated that it is the mislocalization of EGFR itself that results in these events and promotes EGFR-dependent cellular migration. Analysis of patient survival data indicates that these changes may be important indicators of Llgl1's tumor suppressor capability.

Maintenance of cell polarity and tissue architecture are essential in preventing neoplasia. Mammary stem cells are found sporadically throughout the ductal epithelium and help contribute to replenishing lost or dead epithelial cells. These stem cells asymmetrically divide to continue the stem cell line and propagate epithelial cells which differentiate to function as luminal epithelial cells. While exact methods of identifying of these mammary stem cells is still debated, many agree that high CD44 expression, low CD24 expression, low CD49f expression, and the ability to form mammospheres are excellent indicators of individual mammary stem cells [45, 46] and potentially breast cancer stem cells [25]. A knockout of Llgl1 expression in MCF12A cells results in the development of high CD44 expression, low CD49f expression, and increases mammosphere formation. While these changes don't elicit true stem cell qualities, i.e. mammospheres were not formed from a single cell, they do appear to induce a type of metaplasia. Metaplasia is associated with changes in polarity, such as a breakdown of cell-cell junctions, which is a phenotype observed when polarity proteins are lost [47–49].

Polarity proteins play an important role in regulating the protein complexes that form tight, adherence and gap junctions. The apical localization of Par3 initiates cell-cell junctions and it localizes to tight junctions where it, along with the other Par and Crumbs complexes, helps the maturation, formation, and remodeling of these junctions [49]. MCF10A cells do not form tight junctions because they lack Crumbs3 [50] and this loss could account for the differences we observed between MCF10A and MCF12A cells when an additional polarity protein (Llgl1) is lost. In addition to their role in junction formation and stabilization, polarity proteins also impact cellular proliferation and their loss can block apoptosis, when in conjunction with oncogenes, can significantly increase tumor growth and invasion [48]. Of note, increased TAZ has been shown to activate the EGFR pathway, which can then result in a loss of E-cadherin expression at adherence junctions, drive migration, increase CD44 expression, and promote growth in soft agar and mammospheres [30, 31, 36]. It is interesting to note that loss of Llgl1 results in a downregulation of E-cadherin, as well as cell migratory behavior indicative of a loss of cell-cell junctions. Loss of Llgl1 drives these phenotypes in an EGF dependent manner, and alters intracellular EGFR localization and function. Future work will focus on the mechanism by which intracellular EGFR preferentially activates AKT, MAP Kinase and TAZ.

In addition to regulating cell junctions and apoptosis, polarity proteins also control the Hippo pathway. Correct Hippo pathway signaling is important for the proper formation of tissues and organs, including mammary glands. Scribble works within the Hippo pathway to help regulate the signaling and activation of Hippo by binding with Fat and regulating Warts level and stability, and acts as a scaffold to assemble Mst1/2, Lats1/2 and TAZ [16, 51, 52]. In our study, the loss of Llgl1 increases nuclear TAZ but not YAP, a distinction that indicates Llgl1 may selectively impact the Hippo pathway, something future experiments will examine.

We have shown that the loss of Llgl1 also promotes stem-cell like qualities in breast epithelial cells as indicated by the CD44_hi_/CD49f_lo_/CD24_lo_ expression in MCF12A cells. The CD44_hi_/CD24_lo_ lineage specifically correlates with increased migration [53] and distant metastases [54]. In an attempt to address the role of Llgl1 in tumor progression, we transfected an Llgl1-GFP construct into breast cancer cell lines with little to no Llgl1 expression, including MDA-MB-453, MDA-MB-231, and T47D. In each case, these cell lines died within 72 hours of transfection with Llgl1 (in contrast to control-GFP transfected cells which continued to survive, data not shown), which prevented us from performing any long term cell growth or transplant experiments. Increased breast cancer metastases decrease patient survival, a correlation that we also observed with loss of Llgl1 expression. Llgl1's role as a polarity regulator in the Scribble complex is important, and as we have shown, its loss results in an increase of cancer stem-cell like qualities, migration, and transplant survival in an EGF-dependent manner. Our data shows that Llgl1 is a necessary regulator in the prevention of metaplasia and that its loss results in multiple aberrant characteristics, all of which can decrease patient survival, warranting further exploration of Llgl1 as a tumor suppressor and potential therapeutic target.

## MATERIALS AND METHODS

### Tissue culture

MCF10A and MCF12A cells were obtained from ATCC and maintained in DMEM/F12 media, 1% Pen/Strep, 20ng/mL Recombinant Human Epidermal Growth Factor (Corning), 5% Donor Horse Serum (Omega Scientific), 0.5μg/mL Hydrocortisone (Sigma), 100ng/mL Cholera Toxin (Sigma), 10ng/mL Bovine Insulin (Fisher). MDCK II cells were obtained from ATCC and were grown in MEM media, 10% FBS (Corning), and 1% Pen/Strep. Stably transfected MDCKs were grown in complete MEM media as listed previously with 0.5mg/mL G418, 2μg/mL puromycin, and 10ng/mL dox. Before imaging cells were treated with 2mM sodium butyrate for 16 hours. All cells were incubated in 5% CO_2_ at 37°C. Cells grown on plastic were imaged using a Leica DMIL microscope on the 10x objective with a Nikon CoolPix 4500 camera on a 1x C-mount.

### Llgl1 silencing and expression

MCF10A cells were transduced at 12 MOI and MCF12A cells were transduced at 2.5 MOI with MISSION Lentiviral transduction shRNA particles to knockdown Llgl1 expression (Sigma SHCLNV NM_004140 clone TRCN0000117138) or with non-mammalian shRNA control transduction (Sigma SHC002V). Selection of transduced cells was established and maintained by addition of 1μg/mL Puromycin (Fisher).

### EGFR construct transfections

MDCK II cells were double transfected using Clontech Lenti-X Tet-On 3G Inducible Expression System. A pLVX-Tet3G (Cat# 631187) plasmid was transfected into parental MDCK II cells by combining pLVX-Tet3G plasmid with Lipofectamine 2000 in transfection media (MEM +10% FBS +1% P/S). Selection was established and maintained with 0.5mg/mL G418. Once a stable stock was generated the second plasmid pLVX-Tre3G (either an empty vector (EV), EGFR-GFP^WT^, or EGFR-GFP^P667A^) was introduced using the same method. Selection of double transfected cells was established and maintained with 3μg/mL puro in addition to 0.5mg/mL G418. MCF12A cells were transiently transfected using a CMV-EGFR-GFP^WT^ or CMV-EGFR-GFP^P667A^ plasmid with Lipofectamine 2000 and fixed within 48 hours for imaging.

### Western blotting and antibodies

Cultured cells were lysed in ice-cold lysis buffer containing 20mM TRIS pH7.5, 150mM NaCl, 1% NP40, 5mM EDTA pH 8.0, 1% NaF, 1% NaVO_4_, 0.1% NH_4_ Molybate and 8% Complete phosphatase and protease inhibitor (Roche). The lysates were centrifuged and supernatant was collected for Western blot analysis and stored at −80°C. Protein lysate was separated by SDS-PAGE and transferred to PVDF membrane (Millipore). The membrane was blocked in 5% milk in PBS/0.1% Tween solution for Llgl1 antibody, 5% BSA in 10x TBS solution for Integrin α6 antibody, or 3% BSA in TBS/0.1% Tween solution for all other antibodies and then used for immunoblotting. Proteins on the membrane were treated with SuperSignal West Pico Chemiluminescent Substrate (Pierce), visualized on Blue Autoradiography film (GeneMate) and developed with a Konica SRX-101C. Antibodies included Llgl1 (Abnova H00003996-M01), E-cadherin (H-108; Santa Cruz sc-7870), dpERK 1&2 (Sigma M8159), ERK 1&2 (Cell Signaling Technologies 4370), pStat3 (Tyr705; Cell Signaling Technologies 9145), Stat3 (124H6; Cell Signaling Technologies 9139), pAkt (Ser473; Cell Signaling 4060), Akt (Cell Signaling 9272), TAZ (V386; Cell Signaling 4883), Slug (C19G7; Cell Signaling 9585), EGFR 1005 (Santa Cruz sc-03), and βactin (Sigma Aldrich A5441). Integrin α6, was a kind gift from Dr. Anne Cress.

### Fluorescence activated cell sorting and analysis and antibodies

Cells were detached with a 0.025% Trypsin/2.21 mM EDTA solution in PBS, centrifuged and resuspended in cold PBS, and labeled with CD24-FITC (eBioscience 11-0247), CD44-APC (eBioscience 17-0441), and CD49f-PE (eBioscience 12-0495). After labeling, cells were resuspended in 2% PFA for analysis or cold PBS for sorting. Three-color flow cytometric analysis was performed using a FACScanto II flow cytometer (BD Biosciences, San Jose, CA) equipped with an air-cooled 15mW argon ion laser tuned to 488nm. The emission fluorescence of CD24 FITC was detected and recorded through a 530/30 bandpass filter in the FL1 channel. CD49f PE was detected in the FL2 channel through a 585/42 bandpass filter. CD44 APC was detected in the FL3 channel through a 660/20 bandpass filter. List mode data files consisting of 10,000 events gated on FSC (forward scatter) *vs* SSC (side scatter) were acquired and analyzed using CellQuest PRO software (BD Biosciences, San Jose, CA). Appropriate electronic compensation was adjusted by acquiring cell populations stained with each dye/fluorophore individually, as well as an unstained control. Cell sorting was performed with the FACSaria from BD Sciences. Gates were established based on unlabeled and single labeled cell samples. Both analysis and sorting were performed through the Cytometry Core Shared Resource at the University of Arizona Cancer Center.

### Immunofluorescence and antibodies

Cells were fixed with 4% PFA (Santa Cruz), permeabilized with 0.5% Triton X-100, 0.05% Sodium Azide in PBS for 15 minutes on ice, and then blocked with 20% Fetal Bovine Serum (Corning). Primary antibody incubation was overnight in a humidity chamber at 4°C, while secondary antibody incubation was 1 hour in a humidity chamber at room temperature. Slides were mounted with Prolong Diamond Antifade Mountant (Life Technologies P36961) or Prolong Diamond Antifade Mountant with DAPI (Life Technologies P36962). Images were acquired using a Leica DMLB microscope and Leica DFC 310 FX camera mounted on a 1x C-mount using the LAS V4.5 software or using the Leica SP5-III confocal microscope, courtesy of the Imaging Shared Resource at the Arizona Cancer Center. Antibodies included EGFR (clone 225; Millipore MABF120), GFP (Abcam ab13970), TAZ (H-70; Santa Cruz), Slug (G-18; Santa Cruz), YAP (H-125; Santa Cruz), Alexa Fluor 488 and 594 (Invitrogen).

### Mammosphere assays

Mammosphere media was prepared using Mammocult Basal Medium (Stemcell Technologies), Proliferation Supplement (Stemcell Technologies), 0.5μg/mL Hydrocortisone (Sigma), 0.2% Heparin (Stemcell Technologies) and 0.1% Pen/Strep (Corning). All cells were grown at 37°C in 5% CO_2_. Cells were initially harvested from 2D plastic tissue culture after trypsinizing cells and suspending in normal growth media. Cells were pipetted repeatedly and passed through a 25G needle in order to attain a single cell solution. The solution was then gently spun down at 350g and suspended in 1mL Mammosphere media. Cells were plated into 6-well ultra-low attachment plates (Corning) at 40,000cells/well in Mammosphere media. Each well was continually fed with mammosphere media every 3-4 days. Cells were kept in 37°C incubation for 9 days. Primary mammospheres were spun down, trypsinized and separated to single cell suspension. Cells were plated back onto new 6-well ultra-low-attachment plates at the same previous density. Secondary spheres were grown and fed under the same conditions for another 9 days and quantified. The process was repeated for all future passages. At end of each passage, mammospheres were photographed and measured using ImageJ to quantify the number of spheres > 60μM in diameter. Mammosphere formation efficiency (MFE) was calculated as (#of spheres > 60μM / number of cells plated)*100. Alternatively, MCF12A shLlgl1 CD44_hi_/CD49f_lo_ and CD44_lo_/CD49f_hi_ mammospheres were counted by dispersing into a single cell suspension and counted with Trypan Blue. *N* = 3 for each experimental group. Graphical representation used the mean as the center value with error bars representing one standard deviation in each direction. Each experiment was repeated with at least two biological replicates using different transductions.

### Mammosphere staining

For lineage tracing assay, primary mammospheres were allowed to grow following the normal protocol. After 9 days the mammospheres were collected, trypsinized, centrifuged and resuspended, and incubated in either Di-O, Di-I, or Di-D, followed by washes (Invitrogen Molecular Probes). Cells with each stain were then mixed equally and plated for secondary mammosphere growth following normal protocol.

Actin was visualized by fixing mammospheres in 4%PFA, followed by incubation in 0.1% Triton X-100/1% BSA in PBS. Fixed mammospheres were incubated with Alexa Fluor 488 Phalloidin (Life Technologies A12379) and mounted with DAPI (Life Technologies P36962). Mammospheres were imaged using Leica SP5-III confocal microscope, courtesy of the Imaging Shared Resource at the Arizona Cancer Center.

### MTT assays

Cells were plated in 96 well plates and grown for 3 days. Cell survival was calculated in terms of Fold Change in OD from the corrected values at Day 0 to Day 3. The corrected value is determined by subtracting the OD value of a well with no cells that was incubated with the same media/MTT as was added to cell containing well. Fold Change was calculated as (Day3-Day0)/Day0. For MCF10A, *n* = 16 for each experimental group. For MCF12A, *n* = 6 for no ligand treatment and *n* = 3 for EGF treatment for each cell type. Graphical representation used the mean as the center value with error bars representing one standard deviation in each direction. Each experiment was repeated with at least two biological replicates using different transductions.

### Soft agar colony assay

A 1.2% agar solution was mixed with 2x MCF12A media (2x DMEM/F12 media, 10% Donor Horse Serum (Omega Scientific), 2% Pen/Strep and 40ng/mL Recombinant Human Epidermal Growth Factor (Corning), 1μg/mL Hydrocortisone (Sigma), 200ng/mL Cholera Toxin (Sigma), 20ng/mL Bovine Insulin (Fisher), plated into a 6-well plate. A 0.3% agar solution was combined with trypsinized cells in 2x media and plated into each well on top of the hardened agar layer and incubated at 37°C, 5% CO2 for 13 days. Wells were fed every 3 days and imaged before staining. Cells were imaged using a Leica M2FLIII dissection microscope with 0.63x objective at 0.8x zoom with a 0.5x C mount attached to a Leica DCF310 FX camera and acquired with LAS V4.5 software. Images were processed using ImageJ to quantify the number and size of colonies (Threshold: Regular, 5. Radius: min 2, max 50 (auto max).

### Migration assays

Cells were plated to 95% confluency, washed with PBS, scratched with a p200 pipette tip, rinsed with PBS, and then serum free media with or without EGF (20ng/mL) was added back onto the cells. Images were acquired immediately and every 4 hours thereafter. Images were quantified using ImageJ to measure the area of the scratch at each time point and the area of migration was determined by subtracting from the time 0 area. *N* = 4 for each experimental group. Graphical representation used the mean as the center value with error bars representing one standard deviation in each direction. Each experiment was repeated with at least two biological replicates using different transductions.

### Immunofluoresence of cells on transwell filters

MCF12A shControl, shLlgl1, CD44_hi_/CD49f_lo_, and CD44_lo_/CD49f_hi_ cells were grown on 0.4μm pore size polyester membrane transwell filters (Corning) in normal growth media then fixed, permeabilized, and incubated overnight with anti-GM130 (BD Biosciences 610823) and Alexa Fluor anti-mouse 594 (Invitrogen). Filters were mounted with DAPI (Life Technologies P36962). Images were acquired using a Leica SP5-III confocal microscope.

### Mouse experiments

Immunocompromised (NOD-SCID) mice (Taconic, Rockville, MD) were tested for the presence of serum IgG and found to be < 20 μg/ml IgG. Female mice (four to six weeks old) were injected with cells embedded in Matrigel (BD Biosciences) into the mammary fat pad and palpated. Size of masses were determined based on the formula *a*^2^ x *b*/_2_ where *a* is the smaller diameter and *b* is the larger diameter. MCF12A shControl, shLlgl1, CD44_hi_/CD49f_lo_, CD44_lo_/CD49f_hi_ cells were grown on plastic then trypsinized, spun down, and resuspended in matrigel. 500,000 cells were injected into the mammary fat pad of NOD-SCID mice (shControl *n* = 3, shLlgl1 and others *n* = 4). Tumor size was measured using palpation every 3-4 days until undetectable by the Experimental Mouse Shared Resource. This experiment was not blinded but cells were injected into mice chosen at random and masses were palpated and recorded by individual mouse number without indication of cell type except on a master sheet. A biological replicate was performed using a different transduction. For sample size estimate, animal studies estimated an 80% power to detect proportion of survival of at least 21% between any of groups; this is based on a log-rank test statistic (*p* = 0.05).

### Patient survival probability

A breast tumor gene expression dataset (GSE3494) with associated clinical variables was downloaded from GEO (www.ncbi.nlm.nih.geo). Patient expression data was divided into two types of cohorts based on high *vs*. low expression of Llgl1 and elston histological grades (1-3). Analysis of survival time was performed using R statistical software and survival package. Long-term overall survival was analyzed by Kaplan-Meier method. We performed a logrank test using function survdiff to investigate if any, differences between Llgl1 expression groups and elston grades groups on survival time. The p-value was calculated from a chi-square test.

### Significance calculation in all figures

Variance was similar between all groups that were statistically compared. The values for each group were compared statistically using a two-tailed student *t* test with 95% confidence as the cutoff for statistical significance. Increasing levels of confidence are indicated as * = *p*≤0.05, ** = *p*≤0.01, *** = *p*≤0.001, **** = *p*≤0.0001.

## SUPPLEMENTARY MATERIALS FIGURES




