# Supplementary material for: Llgl1 prevents metaplastic survival driven by epidermal growth factor dependent migration
Source: Oncotarget. 2016 Aug 17;7(38):60776–92. doi: 10.18632/oncotarget.11320 (PMC5308616; doi:10.18632/oncotarget.11320)
Supplement: Supplementary file 1 [file oncotarget-07-60776-s001.pdf]

Llgl1 prevents metaplastic survival driven by epidermal growth factor dependent migration

Supplemental Material

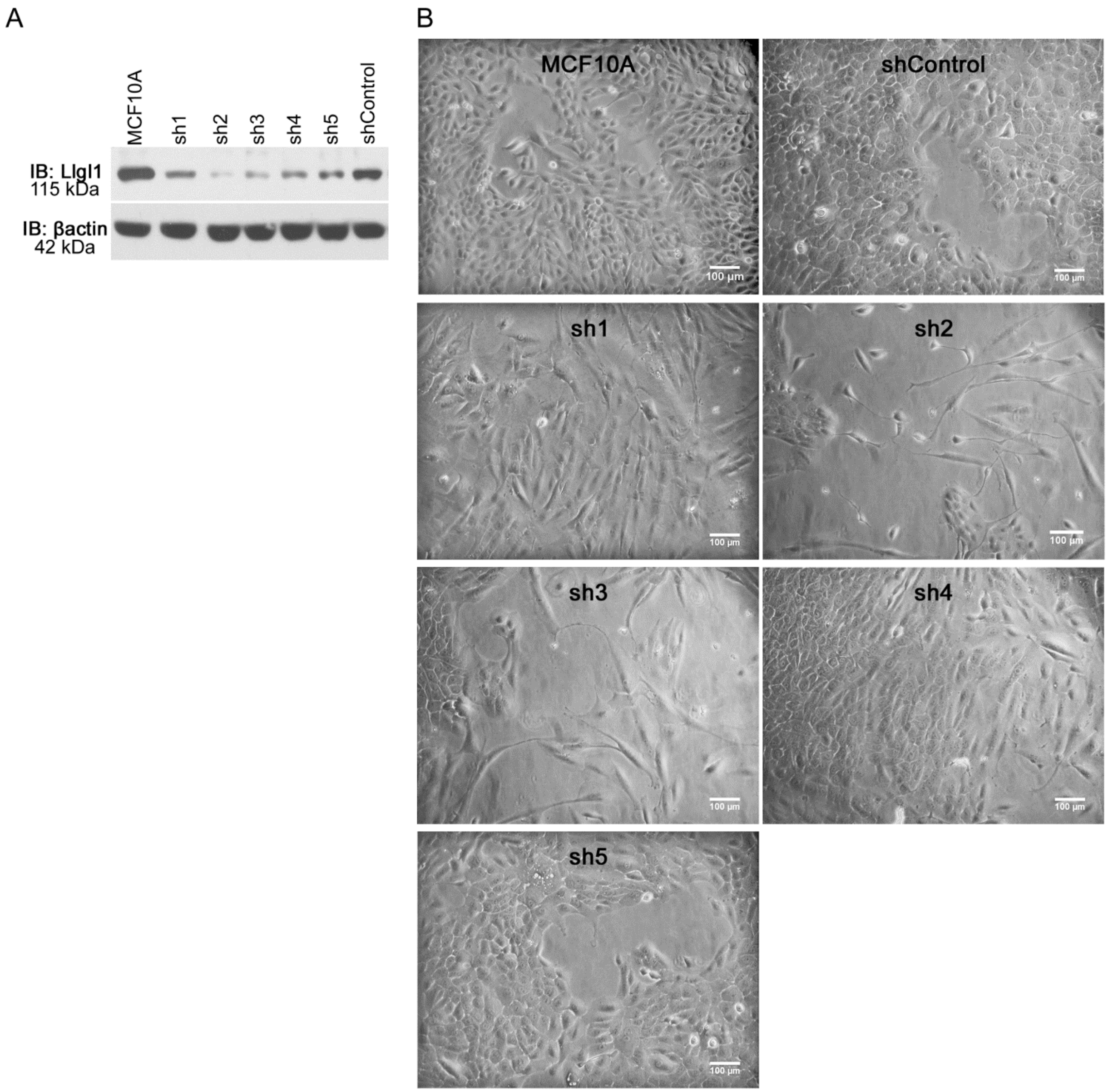

Supplemental Figure 1. Optimization of Llgl1 knockdown using multiple shRNAs targeting Llgl1. MCF10A cells were transduced with 5 different shRNAs targeting Llgl1. (A) Protein

lysates were analyzed by immunoblot using antibodies: anti-Llgl1 and anti-βactin. (B) Cells were grown on plastic and observed for a mesenchymal phenotype.

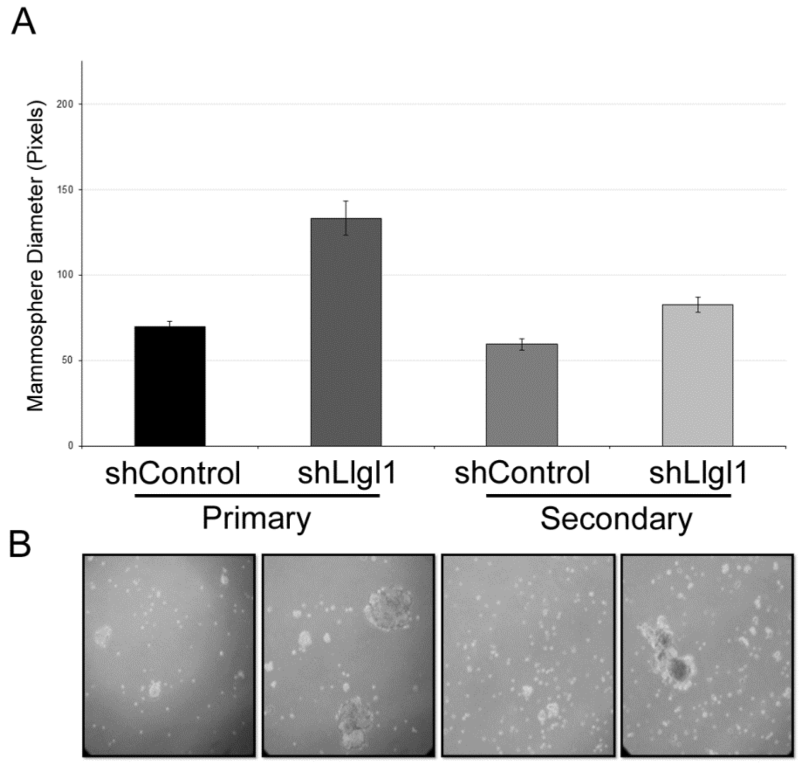

Supplemental Figure 2. Loss of Llgl1 in MCF10As increases mammosphere growth. (A and B) MCF10A shControl and shLlgl1 cells were evaluated for mammosphere growth and loss of Llgl1 resulted in increased mammosphere growth and size in both primary and secondary mammospheres.

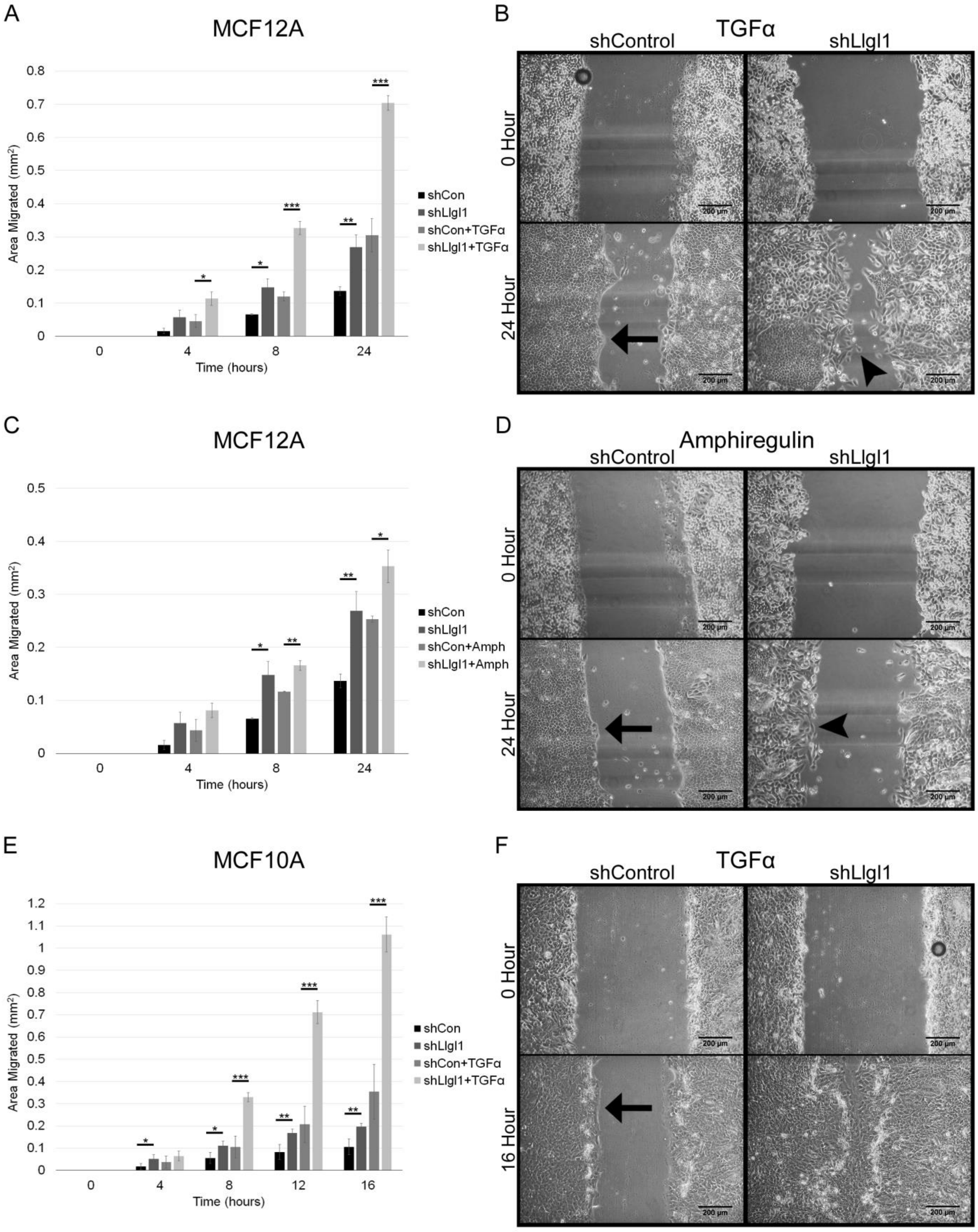
A
MCF12A
Area Migrated (mm²)
0
0.1
0.2
0.3
0.4
0.5
0.6
0.7
0.8
Time (hours)
0
4
8
24
*
*
***
**
***
shCon
shLlgl1
shCon+TGFα
shLlgl1+TGFα
B
TGFα
shControl
shLlgl1
0 Hour
24 Hour
200 µm
C
MCF12A
Area Migrated (mm²)
0
0.1
0.2
0.3
0.4
0.5
Time (hours)
0
4
8
24
*
**
**
*
shCon
shLlgl1
shCon+Amph
shLlgl1+Amph
D
Amphiregulin
shControl
shLlgl1
0 Hour
24 Hour
200 µm
E
MCF10A
Area Migrated (mm²)
0
0.1
0.2
0.3
0.4
0.5
0.6
0.7
0.8
0.9
1
1.1
1.2
Time (hours)
0
4
8
12
16
*
*
***
**
***
**
***
shCon
shLlgl1
shCon+TGFα
shLlgl1+TGFα
F
TGFα
shControl
shLlgl1
0 Hour
16 Hour
200 µm

Supplemental Figure 3. Loss of Llgl1 induces migration in the presence of multiple EGFR ligands. (A-F) MCF12A and MCF10A control and Llgl1 knockdown cells were generated as described in Fig. 1, grown to confluence, scratched, and then observed for wound healing migration in serum free media with either the absence of ligand (A, C, and E) or in the presence of TGFα (20ng/mL) (A, B, E, and F) or the presence of Amphiregulin (20ng/mL) (C and D). Migrating epithelial sheets are indicated by arrow, disorganized cellular groups and single cells are indicated by arrowheads. Error bars show ± standard deviation. * P<0.05, ** P<0.01, *** P<0.001, **** P<0.0001.

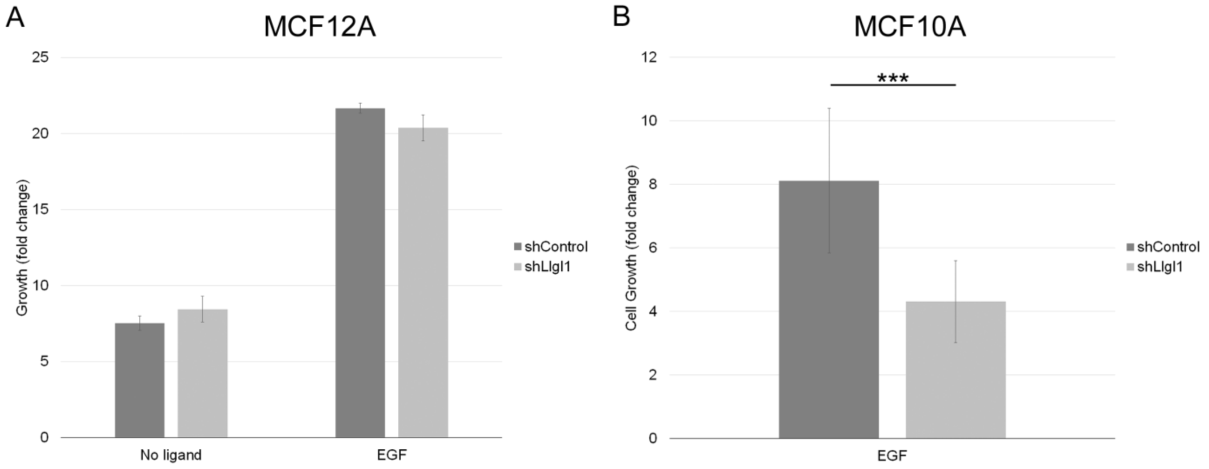

Supplemental Figure 4. Loss of Llgl1 does not increase cell growth with EGF. (A) MCF12A shControl and shLlgl1 cells were grown for 3 days and analyzed by MTT to determine cell growth in the absence or presence of EGF (20ng/mL). (B) MCF10A shControl and shLlgl1 cells were grown for 3 days and analyzed by MTT to determine cell growth in the presence of EGF (20ng/mL).

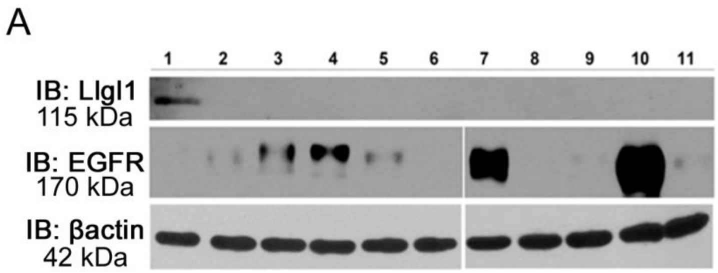

Supplemental Figure 5. Human Breast Tumors have lost Llgl1 expression and have high EGFR expression. (A) Human PDX patient sample lysates were immunoblotted for anti-Llgl1, anti-EGFR, and anti-βactin and showed that most human breast tumors have lost Llgl1 expression and have high levels of EGFR expression.

See online for Supplemental Video

Supplemental Video 1. MCF12A shLlgl1 CD44$_{hi}$/CD49f$_{lo}$ cells migrate randomly. MCF12A shLlgl1 CD44$_{hi}$/CD49f$_{lo}$ cells were plated and grown to confluence under normal growth conditions, scratched, and put in serum-free media with EGF (20ng/mL). After an 8 hour incubation cells were imaged by time-lapse confocal microscopy every 10 minutes for 2.5 hours. Videos of the images were processed, displaying 2 frames/second. Migrating cells indicated by the arrowheads.
